# The Biochemical Detection of Inherited Diseases
*Based on a lecture to the South West Paediatric Club on October 17th, 1965.


**Published:** 1966-04

**Authors:** J. B. Holton

**Affiliations:** Principal Biochemist, Southmead Hospital


### THE BIOCHEMICAL DETECTION OF INHERITED DISEASES*

BY

J. B. HOLTON, Ph.D.

*Principal Biochemist, Southmead Hospital*

### INTRODUCTION

The concept that the primary cause of some inherited diseases was a block or "error" in the metabolism of some specific clinical substance was introduced by Archibald Garrod in 1908; but it was almost half a century before this hypothesis was seen to have very wide application. The rapid growth that then took place in the investigation of inborn errors of metabolism was dependent on the increasingly biochemical approach to medicine, and the production of simple techniques for detecting these disorders. Precise diagnostic tests are now available to the clinician, and rational and successful treatments have been found for some diseases which, 10 to 15 years ago, were regarded as hopeless. For those interested in chemical pathogenesis, the diseases in which the fundamental defect is known appear to provide excellent subjects for study. The problem is to discover how all the signs and symptoms of the disease arise from the one basic alteration in metabolism.

In the following discussion the principles involved in detecting inherited metabolic diseases are considered. A short section deals with biochemical methods of tracing heterozygotes for these conditions which, when successful, can make possible more accurate genetic counselling.

### METHODS OF DETECTION OF INBORN ERRORS OF METABOLISM

In the usual form of inherited metabolic disorder there is a block in the breakdown pathway of a compound, or a failure to convert one substance to another, in the body tissues. This may be illustrated by considering a compound A (this might be glycogen, galactose or an amino acid) which is converted in the tissues to a series of other chemical compounds B, C and D. Thus we can represent the metabolic pathway:

$$A \longrightarrow B \longrightarrow C \longrightarrow D$$

In this scheme B, C and D are metabolic products, or metabolites of A. It is well known that for each step in a metabolic pathway a specific enzyme is required. The absence of one of these enzymes causes a block in the pathway and this is the fundamental defect in an inborn error of metabolism.

There are three methods by which the absence of an enzyme from a metabolic pathway can be detected:

1. The enzyme activity may be demonstrated to be absent by a direct assay procedure. This may only be possible if liver or muscle biopsy material is available and recently some enzymes have been assayed using pieces of jejunal mucosa. However, it is often possible to carry out an enzyme estimation using materials which are obtained with ease. For example, red blood cells may be used to demonstrate the enzyme defect in galactosaemia (Beutler, 1964), maple-syrup urine disease may be detected by using leukocytes (Dancis, Hutzler and Levits, 1963), and the diagnosis of histidinaemia may be established by measuring the activity of the enzyme histidase in skin (Zannoni and La Du, 1963).

* Based on a lecture to the South West Paediatric Club on October 17th, 1965.

2. As a consequence of the enzyme deficiency there may be an accumulation of compounds which precede the block, and substances due to be formed after the block will not be synthesized. If a block occurs between A and B, in our hypothetical pathway, then A should accumulate. This depends on A continuing to be produced from some source, or being absorbed from the gastro-intestinal tract if it is a dietary substance. The amount of A will increase within the cells of the body but we can assume that it will leak out, thus raising the concentration of A in the body fluids. If the substance we are looking for is excreted by the kidneys, urine examination is the obvious choice but, providing it is not cleared completely by the kidneys, the compound will have an increased blood concentration also.

3. When there is an intracellular accumulation of A it may be metabolized along a pathway which it does not usually follow. Thus a series of abnormal metabolites X, Y etc. will be produced:

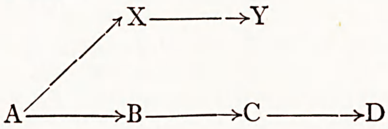

This is the basis of the simple phenistix and ferric chloride tests for phenylketonuria. In this disorder the enzyme which converts phenylalanine to tyrosine is missing and phenylalanine accumulates. This leads to the production of a number of abnormal compounds, one of which, phenylpyruvic acid, gives a positive result when the phenistix and ferric chloride test are performed on urine.

It should be remembered that the production of an abnormal metabolite is several steps removed from the primary enzyme block and so a test based on this principle would be expected to be the least satisfactory of the methods of detection.

Occasionally the effect of the enzyme block can be magnified before applying either tests 2 or 3, by giving a loading dose of A. As a general rule this technique is contraindicated in a patient because A, or its abnormal products, may be toxic.

### METHODS OF DETECTION OF TRANSPORT DISORDERS

The inherited diseases discussed so far are caused by a defect in an intracellular enzyme. There is another important group in which the primary defect is in the mechanism for transporting a substance, or group of substances, across a cellular membrane. These are frequently called transport disorders. The defect may prevent transport across the gut wall, in which case it is apparent because the substance involved will not be absorbed; or it may involve predominantly the renal tubular reabsorption mechanism for a compound. In most diseases both defects occur together.

In the first case, that is, failure of absorption from the gastro-intestinal tract, it may be possible to show an increased concentration of the substance in the faeces. Because of this accumulation of a substance in the gut there could be considerable quantities of abnormal metabolites produced from it by bacterial action. These metabolites may be absorbed and then appear as abnormal compounds in the urine. This is the basis of the finding of high urinary excretion of indolic compounds in Hartnup disease, in which there is a failure to absorb tryptophane from the jejunum. Disorders showing primarily defective renal tubular transport will obviously be identified by increased urinary excretion of the substance, or substances, concerned. Levels of these substances in blood are usually normal or slighly low.

The nature of the transport mechanisms discussed in the preceding paragraphs is not yet understood but the cause of the inherited forms of disaccharide intolerance, which show a similar picture of malabsorption, is clearer. The disaccharides sucrose, lactose, and maltose are hydrolyzed to monosaccharides by enzymes present in the cells of the jejunal mucosa. Hydrolysis of disaccharide molecules has to occur before normal absorption of carbohydrate can take place. In the absence of disaccharidase activity there will be malabsorption of the disaccharide.

Disaccharidase deficiency is demonstrated by finding the corresponding disaccharide in faeces, and frequently a low faecal pH is produced because the sugar is fermented in the gut. When a disaccharide load is given orally there is no increase of its constituent monosaccharides in the blood stream. Although this condition is in effect a transport disorder, it is produced by the absence of an intracellular enzyme, and the diagnosis can be confirmed by estimating disaccharidase activity in biopsy material from the jejunal mucosa.

### DETECTION OF INHERITED DISEASES OF THE NEWBORN

If an inherited metabolic disorder is suspected in an infant this should be investigated with all possible speed because the serious consequences to the developing infant may be averted by appropriate treatment. There are three detection problems in this period which will be considered separately, since they demand different biochemical approaches:

1. The simplest situation is the investigation of an infant with a family history of a recognized biochemical abnormality which is amenable to treatment. The chance of a positive finding in these circumstances is usually 1 in 4, and so it is worth while using the most sophisticated technique available for the detection of this particular disease. If a reliable enzyme test is available using blood cells this can be carried out on cord blood to get the earliest possible diagnosis.

An interesting development in this field is the attempt by Jeffcoate, Fliegner, Russel, Davis and Wade (1965) to see whether a foetus affected with adrenogenital syndrome has abnormal compounds in the amniotic fluid. The results in two cases suggest that the presence of a 21-hydroxylase defect in the foetus leads to accumulation of pregnanetriol in the liquor amnii. The findings will have to be confirmed by other groups before the technique becomes established, but the principle of amniotic fluid examination for the ante-natal diagnosis of metabolic disorders might have much wider applications.

2. The biggest detection problem is the screening programme for the whole newborn population. The metabolic disorders to look for are those which are most common and which can be treated most successfully. At the same time the cost and practicability of any scheme have to be taken into account. A committee of the American Academy of Pediatrics (1965) which considered these questions recently concluded that a minimum of two tests should be carried out; a blood phenylalanine test to detect phenylketonuria, and a test for urine reducing substances to detect, principally, galactosaemia. They suggested that these should be performed at about the end of the first and fourth weeks of life.

The emphasis in screening is now on very early detection and it is realized that the urine phenistix and ferric chloride tests for phenylketonuria are unsuitable for this (Efron and MacCready, 1964). The blood phenylalanine methods which are most appropriate for mass screening purposes are the bacteriological procedure (Guthrie and Susi, 1963) and a fluorimetric technique (McCaman and Robins, 1962) which Hill, Summer, Pender and Roszel (1965) have recently adapted for the auto-analyser. Heel-prick blood for these phenylalanine methods can be transported in capillary tubes or on filter paper. The test appears technically straightforward but the large-scale collection of blood, particularly from babies born at home, would probably present some difficulties.

Trial surveys to screen for a large number of inherited disorders are obviously desirable, and give valuable information. In Manchester six metabolic diseases (phenylketonuria, homocystinuria, glycinuria, maple-syrup urine disease, tyrosinaemia and histidinaemia) are being looked for by paper chromatography carried out on capillary blood collected between the 6th and 26th days of life (Komrower, 1965).

3. The final problem in the neonatal period is that of the babies who fail to thrive. The difficulty here is the large number of biochemical abnormalities which produce very similar clinical signs. If only the amino acid disorders are considered (Table I), about 20 of these (shown with an asterisk in the Table) have been found in association with brain damage. Most of these could produce the picture of failure to thrive with convulsions.

TABLE I

Amino acid disorders

1. *Inborn errors of metabolism with high plasma and urine levels of amino acids*

| | |
|---|---|
| *Phenylketonuria | *Hydroxyprolinaemia |
| Tyrosinosis | *Homocystinuria |
| *Histidinaemia | *Hyperlysinaemia |
| *Maple-syrup urine disease | *Citrullinaemia |
| *Hypervalinaemia | *Congenital lysine intolerance |
| *Hyperglycinaemia | *Oasthouse urine disease |
| *Hyperprolinaemia | |

2. *Inborn errors of metabolism involving compounds with no tubular reabsorption showing only high urine levels of amino acids.*

| | |
|---|---|
| *Arginosuccinicaciduria | Hypophosphatasia |
| *Cystathioninuria | β-Amino-isobutyric aciduria |
| *Homocystinuria | *Juvenile amaurotic familial idiocy |

3. *Transport disorders producing amino-aciduria*

| | |
|---|---|
| Cystinuria | *Methionine malabsorption |
| *Hartnup Disease | Glycinuria |
| *Joseph's Syndrome | |

The most helpful method of investigating this group of babies is to carry out the widest range of biochemical tests possible. Fortunately, all the amino acid disorders can be detected by the use of paper chromatography, or a similar technique, on a urine sample. Other abnormal urinary metabolites may be revealed by applying suitable staining procedures to the same paper chromatograms. Simple tests can be performed routinely on urine to detect a number of other disorders. The types of test which may be done are those used by Carson and Neill (1962) in their survey of mental defectives in Northern Ireland. For a variety of reasons, biological and technical, biochemical signs may be missed on one urine specimen. It is much better to send urine specimens to the laboratory on several occasions.

### THE DETECTION OF BIOCHEMICAL DISORDERS IN THE MENTALLY DEFECTIVE

Not all patients with inherited disease have severe manifestations in infancy, and many may present first with mental retardation at a later age. It is becoming a common practice to examine all patients with mental retardation for the presence of a metabolic disorder. With some patients there may be features which suggest a specific disease, and thus a special line of investigation, but in most cases one can only carry out similar tests to those described for the "failure to thrive" babies.

The realization that phenylketonuric mothers produce mentally retarded offspring is very important in considering the cause of mental disease. Some individuals may be relatively mildly affected by this disease and a few affected women are known to have married and produced children (Mabry, 1963). Although these children were not phenylketonurics they have proved to be very badly affected mentally, and it is suggested that this is because they have been exposed to a high phenylalanine level *in utero*. It is probable that other conditions may have similar results. As a logical consequence of this finding, metabolic errors should be looked for in the mother when none is found in the affected child.

Screening the mental defective population by means of paper chromatography, and other tests, is most useful in indicating the relative incidence of different disorders in particular areas and may discover new diseases (Carson and Neill, 1962). Alone, these surveys can give false ideas about the association of a particular disorder with mental disease. For example, surveys of mentally defective populations usually detect a few cases of cystinuria. However, because we know that cystinuria frequently presents with renal stones we have a means of suspecting it in otherwise normal individuals, and thus its incidence in mental defectives is not thought to be any higher than in the population as a whole.

### THE PROBLEM OF DETECTION OF HETEROZYGOTES

Most metabolic diseases have a recessive pattern of inheritance. Although the full clinical picture of the disease is only manifest in abnormal homozygotes, the possession of one abnormal gene can sometimes be shown biochemically. Sometimes limiting values for a suitable biochemical parameter can be established for abnormal homozygotes, heterozygotes (parents of children with the full clinical disorder) and the normal population. In the ideal case there should be no overlap in the range for normals and heterozygotes, so that these genotypes could be clearly distinguished. In practice this state of affairs is rarely encountered, but quite a number of conditions exist in which the genotype of the relatives can be determined with sufficient reliability to be extremely valuable for the purposes of genetic counselling.

The principles used in detecting heterozygotes are the same as those elaborated for the detection of the disease, but one is looking for a partial reduction rather than for a complete absence of enzyme activity. The enzyme itself can only be assayed if a method is available on an easily accessible tissue. This technique has been successfully applied to erythrocytes in galactosaemia (Walker, Hsia, Slatis and Steinberg, 1962), to leukocytes in maple syrup urine disease (Dancis, Hutzler and Levitz) and to the skin in histidinaemia (Holton, 1965).

The accumulation of normal and abnormal metabolites, and their estimation in blood and urine, does not often produce a clear difference between normals and heterozygotes. Important exceptions to this rule have been demonstrated in gargoylism, in which carrier relatives excrete a significantly increased amount of chrondroitin sulphate B (Teller, Rosevear and Burke, 1961) and in one type of cystinuria in which heterozygotes have abnormally high concentrations of cystine and lysine in their urine (Harris, Mittwoch, Robson and Warren, 1955). The "loading test", which is designed to increase the accumulation of a particular metabolite artificially, is probably more suitable for the detection of heterozygotes than for detecting those with the disease. The most successful method of detecting carriers for phenylketonuria measures blood concentrations after an oral dose of phenylalanine (Renwick, Lawler and Cowie, 1960).

### REFERENCES

American Academy of Pediatrics (1965). Committee report on the screening of newborn infants for metabolic disease. *Pediatrics* **35,** 499.

Beutler, E., Baluda, M. and Donnell, G. N. (1964). *J. Lab. clin. Med.* **64,** 694.

Carson, N. A. J., Neill, D. W. (1962). *Arch. Dis. Childh.* **37,** 505.

Dancis, J., Hutzler, J. and Levitz., M. (1963). *Pediatrics* **32,** 234.

Dancis, J., Hutzler, J. and Levitz. M. (1965). *J. Pediat.* **66,** 595.

Efron, M. L. and MacCready, R. A. (1965). *J. Am. med. Ass.* **191,** 391.

Guthrie, R. and Susi, A. (1963). *Pediatrics* **32,** 338.

Harris, H., Mittwoch, U., Robson, E. B. and Warren, F. L. (1955). *Ann. hum. Genet.* **20,** 57.

Hill, J. B., Summer, G. K., Pender, M. W. and Roszel, N. O. (1965). *Clin. Chem.* **11,** 541.

Holton, J. B. (1965). *Clin. chim. Acta.* **11,** 193.

Jeffcoate, T. N. A., Fliegner, J. R. H., Russel, S., Davis, J. C. and Wade, A. P. (1965). *Lancet* **2,** 553.

Komrower, G. M. (1965). Personal communication.

Mabry, C. C., Denniston, J. C., Nelson, T. L. and Son, C. D. (1963). *New Eng. J. Med.* **269,** 1404.

McCaman, M. W. and Robins, E. (1962). *J. Lab. clin. Med.* **59,** 885.

Renwick, J. H., Lawler, S. and Cowie, V. A. (1960). *Amer. J. hum. Genet.* **12,** 287.

Teller, W. M., Rosevear, J. W. and Burke, E. C. (1961). *Proc. Soc. exp. Biol.* (N.Y.) **108,** 276.

Walker, F. A., Hsia, D. Y-Y., Slatis, H. M. and Steinberg, A. G. (1962). *Ann. hum. Genet.* **25,** 287.

Zannoni, V. G. and La Du, B. N. (1963). *Biochem. J.* **88,** 160.

