# Corrigenda

**Published:** 1966-07

**Authors:** 


					Corrigenda :
Issue of July 1966.
2. Page 43. The lower diagram corresponds to
the legend of Fig. 1, and the upper diagram
to the legend of Fig. 2.

				

